# Parental Expressed Emotion and Behavioural Outcomes in Autistic Children and Adolescents: A Systematic Review

**DOI:** 10.1007/s10578-023-01660-4

**Published:** 2024-02-06

**Authors:** Corinne Marshall, Rosa Hoshi, James Gregory

**Affiliations:** 1 South Wales Doctoral Programme in Clinical Psychology, Cardiff University, Cardiff, UK; 2 Cardiff University, 70 Park Place, Tower Building, 11th Floor, Cardiff, CF10 3AT UK

**Keywords:** Expressed emotion, Behavior, Autism, Parent–child relationship

## Abstract

Growing interest in the links between parent–child relationships and child behavioural presentations in families of autistic children has led to an increased use of the Five Minute Speech Sample (FMSS) measure of parental expressed emotion (EE) in autism research. This review focuses on studies exploring the relationships between parental EE and behavioural outcomes in autistic children. Electronic searches of six databases and grey literature wielded eight studies that met eligibility criteria. Study designs were a mixture of cross-sectional and longitudinal and quality of studies was variable. Parental criticism was largely positively related to, and showed some predictive value for, child behaviour problems. Warmth was mostly negatively related to, and showed some predictive value for, child behaviour problems. Preliminary evidence from one study showed paternal warmth to be significantly related to child behaviours, whilst child behaviours were also significantly related to paternal warmth, suggesting a bidirectional relationship. Analysis of additional EE components produced variable results, however parental stress and depressive symptoms were consistently related to child behaviour, and preliminary evidence suggests a possible role of maternal education level and family cohesion. Outcomes were variable across FMSS coding systems and greater consistency in their application is needed in future research. The current findings suggest that parental EE has an important relationship with child behaviour and future intervention efforts may benefit from aiming to reduced EE in order to improve child outcomes.

## Introduction

It is well-established that parent–child interactions have a significant impact on child well-being and functioning throughout their childhood, adolescence and later life [1, 2]. More recently, a growing body of evidence suggests that it is not only the interaction the parent has with their child, but the attitudes they hold towards their child, such as their expressed emotion (EE) when speaking about their child, that can have this lasting impact [3]. Parental EE was originally measured through the use of the Camberwell Family Interview [4] however this approach was often time-consuming, therefore alternative methods of measuring EE were developed including self-report measures such as the Family Attitudes Scale [5], and briefer interview methods such as the Five Minute Speech Sample (FMSS). The FMSS was originally designed as a brief method of assessing EE in parents of adult children with mental health conditions [6], however, in recent years it has been used to explore EE in a range of family relationships [7]. The FMSS offers a valid alternative method to lengthy observations of parent–child interactions as scores on the FMSS have been found to be associated with parental behaviours and emotions observed in actual parent–child interactions [8]. Parents are asked to talk about their child and their relationship for five minutes and their responses are coded for a range of dimensions of interest. The most commonly used coding system is Magaña-Amato’s [9] EE system, which explores parents’ criticism, hostility, warmth, positive comments, and emotional over-involvement.

High parental EE has been linked to a range of child difficulties, such as behavioural problems and impairments of executive functioning, in typically developing children [10, 11] and increased symptoms in children with mental health conditions, such as depression and anorexia nervosa [12, 13] and physical health conditions such as epilepsy [14]. A growing body of research is evidencing the impact of parental EE on child behavioural and emotional outcomes in children and adolescents with and without mental health and neurodevelopmental conditions and a recent meta-analytic review of 42 studies found a small but significant relationship between maternal criticism and child internalising and externalising problems [15], however this review did not differentiate between typically and atypically developing young people.

Children and adolescents with neurodevelopmental conditions may have greater vulnerability to parental EE, as they are more likely to have impairments in executive functioning impacting their ability to regulate their behaviour, and their parents are more likely to adopt permissive or authoritarian parenting styles [16]. When exploring parental EE using the FMSS, Peris and Hinshaw [17] found that, in school-aged children with ADHD, expressions of aggression were associated with high parental EE, particularly in families which displayed high levels of criticism towards the child. Similar findings were made by Greenberg et al. [18] where high levels of parental criticism were related to high levels of externalising behaviours in individuals with Fragile X syndrome, suggesting that parental EE may impact child externalising behaviours across neurodevelopmental conditions.

Families of autistic children may be particularly vulnerable to these difficulties as around 1 in 2 autistic children have co-occurring emotional and behavioural problems [19], and their parents are more likely to experience higher levels of parenting stress [20] and major or minor depression [21]. Given that, in typically developing children, high levels of parenting stress and low parental psychological wellbeing has been associated with critical parenting behaviours [22], it is likely that similar associations may occur in families of autistic children. Whilst there is some evidence towards these associations, the current literature is not conclusive. For example, Osborne and Reed [23] found that high baseline parenting stress was predictive of lower involvement or poorer communication at follow-up, and Boonen et al. [24] found that higher parenting stress was associated with greater use of harsh punishment and material rewarding along with more negative and criticising interactions among mothers of school-aged autistic children. Meanwhile, Madarevic et al. [25] found no evidence for relationships between negative parenting behaviours and being the parent of an autistic child, therefore further investigation into the relationship between parenting behaviours and interactions within families of autistic children may be beneficial in providing additional evidence in understanding the lives of autistic children and their families.

Recent research has begun to explore the links between parental EE, as measured by the FMSS, and the behavioural and emotional presentation of autistic individuals, with similar patterns of associations being observed as those identified in prior research with other population groups. Woodman et al. [26] explored the trajectory of change in behaviours of adolescents and autistic adults across 8.5 years and found that internalised, externalised and asocial maladaptive behaviours tended to either decrease or remain stable over time as young people transition into adulthood. When exploring FMSS EE components, the quality of mother–child relationship was a significant predictor of behavioural presentation, with higher maternal praise predicting fewer asocial and total maladaptive behaviours at the end of the 8.5 year period. Increases in maternal praise across the course of the study were also found to be associated with reduced externalising and total maladaptive behaviours. Follow-up research supported these findings, with a similar pattern of association occurring whereby mothers who made more positive remarks or displayed higher warmth were significantly more likely to have children who followed a positive trajectory of improvement in behavioural presentation as they transitioned into adulthood, however the impact of maternal criticism on the child’s trajectory was inconsistent across studies [27, 28].

Romero-Gonzalez et al. [29] conducted a systematic review drawing upon eleven studies which explored the relationship between parental EE and psychopathology in autistic individuals. Of these eleven studies, nine utilised the FMSS, whilst two studies used a self-report questionnaire measure of parental EE. Their review concluded, contrary to prior studies, that high levels of EE and criticism, rather than low parental warmth, were associated with externalising behaviour problems in autistic individuals The results of three longitudinal studies included within the review suggested a contrary trajectory to previous studies, revealing a pattern of high levels of criticism and EE predicting subsequent increases in behavioural problems, whilst finding little evidence of a longitudinal impact of warmth on behavioural outcomes. The findings of this review are somewhat limited as four of the included studies drew their data from the same target sample of a larger longitudinal study [30], whilst another two included studies drew from non-independent samples. Despite the differences in evidence regarding specific EE components, parental EE has been shown to be reliably associated with behavioural problems in autistic individuals.

A consistent limitation across these studies exploring the relationship between parental EE and behaviour presentations of autistic individuals, is the wide age range of the participants involved, with many focusing on both adolescent and adult children. Evidence suggests that behavioural and emotional problems decrease as autistic individuals age [31], therefore the inclusion of adults in the sample may increase heterogeneity of scores and may limit what conclusions can be made about supporting individuals during critical developmental periods in childhood and adolescence. Whilst a large proportion of recent research has focused on early child development stages and social, emotional and behavioural outcomes in later life, there is evidence to suggest that the experience of autistic individuals during middle childhood and adolescence can also have an important impact on the development of adaptive behaviours and social skills and therefore the overall quality of life for autistic individuals and their families [32–34]. It would be beneficial to understand the relationship between parental EE and child behaviour problems specifically during childhood and adolescence in order to consider what interventions may be suitable during this period. By understanding these relationships, and the direction of their associations, we may be able to identify interventions that may improve parental EE towards their autistic children such as skills training [3], or management of behaviour difficulties through supporting the increased use of adaptive behaviours in autistic children and adolescents such as early intensive behavioural intervention [35]. Such interventions could potentially also have secondary outcomes of reducing parenting stress and improving psychological wellbeing.

The current review aims to explore the current literature regarding relationships between parental EE, as measured by the Five Minute Speech Sample, and behavioural and emotional outcomes of autistic children and adolescents, and how these relationships may inform suitable interventions to improve the lives of autistic children and adolescents and their families. Through this investigation the review aims to establish whether interventions are likely to be most effective when working directly with autistic children and adolescents, their parents, or the parent–child relationship as a whole.

## Methods

### Search Strategy

This review has been informed by the PRISMA guidelines for reporting systematic reviews [36]. The protocol has been published on PROSPERO (ID: CRD42022315911). A systematic search of articles published prior to 26th April 2022 was conducted across six electronic databases (PubMed, PsycINFO, Scopus, ERIC, ASSIA, and Web of Science). The search terms were limited to variations of 3 key words (“expressed emotion” or “five minute speech sample” and “autism spectrum disorder”) to ensure all relevant papers were identified. The following search terms were mapped to subject headings and keyword terms located in the title, abstract, or key concepts: “expressed emotion” OR “five minute speech sample” OR “5 min speech sample” OR “FMSS” AND “autis*” OR “aspergers” OR “ASD”. The search strategies were reviewed by an experienced subject librarian.

#### Citation Searching

Backward and forward citation searching was undertaken. Reference lists of all included articles were manually screened to identify potential additional studies. Forward citation searching was conducted by searching each included article via Google Scholar and manually screening articles which has cited the included study. This search was initially conducted on 3rd July 2022 and was re-run on 21st April 2023, no additional eligible literature was identified.

#### Grey/Unpublished Literature

Attempts were made to obtain any grey or unpublished literature by contacting prominent authors who have published research into outcomes of parent–child relationships and presentations of autism. None of the contacted authors were able to provide any grey or unpublished literature.

### Inclusion and Exclusion Criteria

Articles were screened utilising the same inclusion and exclusion criteria at each stage of the screening process. Any articles which could not clearly be identified as included or excluded at the title and abstract stage were included within the full-text screening stage to ensure no potential articles were missed.

Articles were included if they fit the following criteria: (a) their participants were parent–child dyads where the child was under the age of 18 and had a diagnosis of autism; (b) the diagnosis had been established using the ADOS, ADOS-2 or ADI-R; (c) the FMSS was used to measure parental EE; (d) the child’s emotional or behavioural presentation was measured; (e) a direct analysis was conducted into the relationship between parental EE and the child’s behavioural or emotional presentation. No restrictions were placed on the measures of child emotional or behavioural measures. Unpublished dissertations were included as long as they were clearly empirical in their approach. Studies published in languages other than English were excluded.

### Search Results

**Fig. 1:**
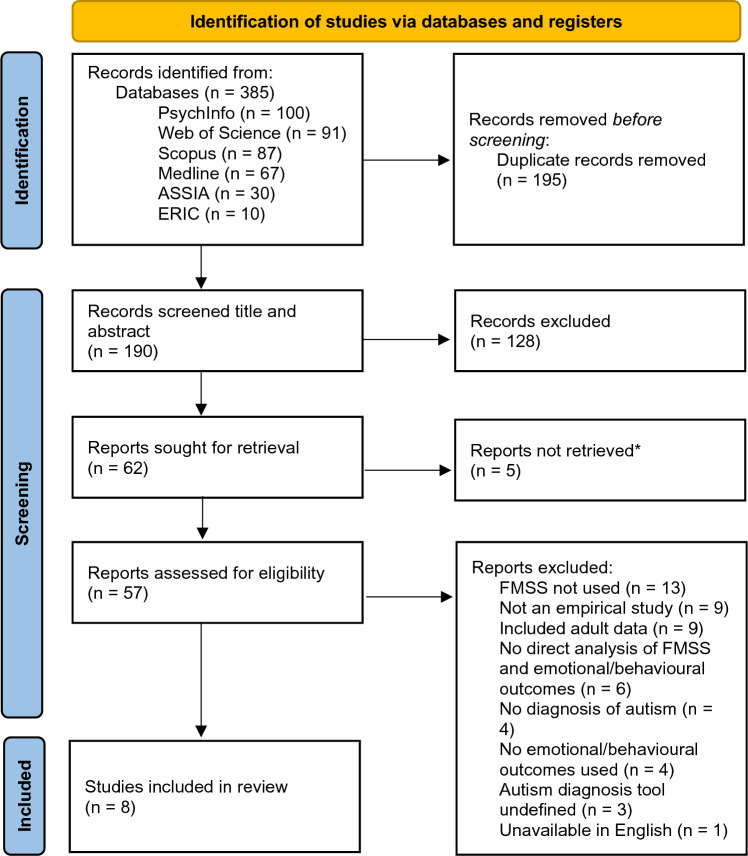
PRISMA flow diagram of the screening process

The initial systematic search results were exported to the reference management software EndNote 20, and following removal of duplicates, 190 articles were identified. Following screening for inclusion and exclusion criteria of title and abstracts, 62 articles remained. Of these 62 articles, it was not possible to retrieve the full-text for 5 articles, therefore 57 full-text articles were screening for inclusion and exclusion criteria. The 5 articles which were not retrieved were posters from conferences and meeting proceedings which were not published as full-text articles. 13 articles were excluded due to there being no use of the FMSS to assess parental EE. 9 articles were excluded due to not being empirical studies. 9 articles were excluded as they included data from children over the age of 18. 6 articles were excluded due to there being no direct analysis of the relationship between parental EE and child behavioural or emotional outcome measures. 4 articles were excluded due to there being no clear diagnosis of autism. 4 articles were excluded due to there being no measurement of child behavioural or emotional presentation. 3 articles were excluded due to the method of diagnosing autism being undefined. 1 article was excluded due to the full-text being unavailable in English. 8 articles were found to fully meet the inclusion criteria (see Fig. 1 for details of the screening process).

#### Data Extraction

A data extraction table was developed and piloted with 2 papers to ensure the table was fit for purpose. Data extraction was completed prior to quality assessment to blind the researcher to the quality of each study and reduce bias in extraction [37].

#### Quality Assessment

Quality assessment of the included articles was completed using the NIH Quality Assessment Tool for Observational Cohort and Cross-Sectional Studies (NIH, [38]). This tool consists of 14 questions that can be answered “yes”, “no”, “cannot determine”, “not applicable”, or “not reported”. This tool aims to assist reviewers in focusing on concepts that are key to a study’s internal validity. Additional areas that may reflect bias within the publications are also noted by the reviewers. Overall quality scores were determined through critical appraisal of responses on the tool, and additional areas of potential bias. The overall quality scores provide a comparison of quality between the included studies and give an indication of how each study should be weighted within the results of the narrative synthesis.

#### Inter-rater Reliability

A second rater was used at all stages of screening, data extraction, and quality appraisal. The second rater screened 40% of the papers for relevant titles and abstracts resulting in an inter-rater agreement level of 89.5%, *k* = 0.78. All disagreements between raters were discussed and a consensus was agreed upon. At the full text-article stage the second rater screened 40% of papers, resulting in an inter-rater agreement level of 100%, *k* = 1.00. At the data extraction stage 50% of included papers were also review by the second rater and an inter-rater agreement level of 100% was achieved, *k* = 1.00. Finally, 50% of included papers were also quality appraised by the second rater, with an inter-rater agreement level of 100%, *k* = 1.00.

## Results

### Study Characteristics

The eight included studies were carried out between 2010 and 2022. Five studies were cross-sectional designs [39–43], and three were a longitudinal design [44–46]. Six studies [39–41, 43–45], were conducted in the USA, one in Israel [42], and one in Australia [46]. Five studies recruited participants from local schools and specialist services [40, 41, 43–45], one study recruited from local service providers and community events [39], one recruited from local services and through media networks [42], one study recruited from a larger longitudinal study but did not provide further detail [46] (Table 1).

**Table 1:** Methodological characteristics of studies

First author (year); recruitment source; location	Study aims and design	Participants	Autism diagnosis tool	FMSS Variable	Child/ adolescent emotional/ behavioural measure	Additional measures	Quality appraisal rating
Baker et al. [39], recruited through flyers at local service providers and community events; USA	Examine associations between parental criticism and behavioural problems in autistic children Cross-sectional	N = 46 children and their primary caregivers; mean age 6.48 years, range 4–11; 78% of children male, 48% Caucasian	ADOS-2	AFMSS + FMSS-EE: criticism and warmth	Child Behaviour Checklist (CBCL)	Child IQ using Stanford-Binet 5 Abbreviated Battery IQ (ABIQ); Child autism characteristic severity using ADOS-2 comparison score	Fair
Benson et al. [40], recruited from public and private schools, multi-system special needs programs, and autism service organisations; USA	Examine the reliability and validity of the AFMSS and its associations with child social competence and behaviour problems Cross-sectional	N = 104 children and their mothers; mean age 8.6 years, range 6–9; 86% of children male, 83% Caucasian	ADI-R	AFMSS: all components	Nosinger Child Behaviour Rating Form (NCBRF)	Mother’s depression using Center for Epidemiological Studies-Depression Scale (CES-D)	Fair
Hickey et al. [41], recruited through fliers posted at autism clinics and in community settings, mailings to schools, and research registries; USA	Determine how the emotional quality of family subsystems combine to create various classes of family emotional climate and to identify predictors of class membership Cross-sectional	N = 148 children and their caregivers, mean age 9.05 years, range 6–13 years; 86% of children male	ADOS	FMSS-EE: criticism and warmth	Child Behaviour Checklist (CBCL)		Good
Hickey et al. [44], recruited through mailings to schools and childcare programs, fliers posted at autism clinics and community settings, and mailings to families in an autism research registry; USA	Examine the bidirectional associations between the emotional quality of parent–child relationships and severity of autism characteristics and emotional and behavioural problems in autistic children Longitudinal	N = 159 children and their caregivers, mean age 9.07 years, range 6–13 years (at Time 1), 86.2% male, 76.7% white non-Hispanic	ADOS	FMSS-EE: criticism and warmth	Child Behaviour Checklist (CBCL) – teacher form	Severity of autism characteristics using Social Responsiveness Scale (SRS-2)	Good
Hickey et al. [45], recruited through mailings to school and childcare programs, fliers posted at autism clinics and in community settings, and research registries; USA	Examine actor and partner effects of level of parenting stress and depressive symptoms on the emotional quality of parent–child relationship in the context of child autism Longitudinal	N = 150 children and their caregivers, mean age = 7.97 years, range 5–12 years, 85.7% of children male, 85% white non-Hispanic	ADOS	FMSS-EE: criticism and warmth	Child Behaviour Checklist (CBCL)	Parenting stress through the Burden Interview, parental depressive symptoms through the Centre for Epidemiological Studies Depression Scale (CES-D)	Poor
Serur et al. [42], children with 22q11DS and autism recruited from a specialist medical centre, TD children recruited via an advertisement in media networks; Israel	Examine associations of parenting stress and parents’ EE with children’s behavioural problems in the context of young children with 22q11DS, compared to children with autism and TD children Cross sectional	N = 74 children and their primary caregivers, child age 3–8 years; 22q11DS = 24, mean age = 5.98 years, 70.8% male; Autism = 28, mean age = 5.35 years, 78.6% male; TD = 23; mean age = 5.48 years, 63.6% male	ADI-R and ADOS-2	Preschool FMSS: criticism and emotional over-involvement	Child Behaviour Checklist (CBCL; Hebrew version)	Parenting Stress Index (PSI; Hebrew version)	Poor
Smith et al. [46], recruited from a larger longitudinal study of autistic children and their families; Australia	Compare the FMSS and AFMSS and investigate their predictive value for concurrent and subsequent child internalising and externalising behaviour problems Longitudinal	N = 51 children and their primary caregivers; mean age = 2.9 years, range 1–3 years; 84.3% of children male	ADOS-2	AFMSS: all components & FMSS-EE: all components	Child Behaviour Checklist (CBCL; 1.5–5 year version)	Parental psychopathology using Depression Anxiety Stress Scale (DASS); maternal education level; child autism severity from ADOS-2 Calibrated Severity Scores; child cognitive ability using the MSEL Early Learning Composite	Good
Zahka [43], recruited from local university centre for autism and related disabilities database, TD children recruited from local public schools; USA	Examine the relationships of family cohesion and EE with child internalising and externalising behaviours, parental beliefs about controllability, and parental stress in sample of high functioning autistic (HFA) children and a matched neurotypical comparison sample Cross sectional	N = 99 children and their primary caregivers; child age range 9–16, mean age 12.9 years; HFA = 56, 89% male; comparison group = 43, 87% male	ADOS-2	FMSS: overall EE	Behaviour Assessment System for Children – Parent Rating Scales (BASC2 PRS)	Wechsler Intelligence Scale for Children (WISC-IV); family cohesion using the Cohesion scale of the Family Environment Scale (FES)	Poor

### Participant Characteristics

The number of participants in study samples ranged from 46 to 159 children/adolescents and their caregivers. The child/adolescent participants ranged between 1 and 16 years old, with the majority of studies having a range of 5 years between their youngest and eldest participants. Mean ages of the children/adolescent participants ranged between 2.9 and 12.9 years old. In all included studies the majority of child/adolescent participants were male, with the proportion ranging from 63.6% to 89%. Five studies recruited one parent [39, 40, 42, 43, 46], with three of these studies including fathers who made up 1–5% of the study samples [39, 42, 43], whilst three studies recruited both parents [41, 44, 45]. Six studies reported the ethnicity of participants [39–41, 43–45] with most of these reporting a majority of Caucasian or non-Hispanic White participants (48–85%). Only three studies reported the details of other ethnicities of participants [41, 43, 45] which included African American (0.7–5%), Hispanic White (8–23%), American Indian (0.7–1%), Asian American (13%), Asian or Pacifier Islander (3%), multiple (1.2–3%), and other (13%) (Table 1).

### EE Measures

Several different approaches currently exist to measure EE within Five Minute Speech Samples and have been used across the included studies (Table 1). One study [43] used Magana et al.’s original coding system [6] (FMSS-EE) which rates four main components: (1) parents’ Criticism, (2) Emotional Overinvolvement (EOI), (3) the quality of their Initial Statement, and (4) the quality of their Relationship. Criticism is established through a frequency count of critical statements, whilst EOI is determined from a range of subcategories including self-sacrificing/over-protective behaviour, emotional display, excessive detail, statements of attitude, and positive remarks. The quality of Initial Statement and Relationship are rated as positive, neutral, or negative. Overall EE status is determined by a combination of these four main components: High EE (Critical) would require either a high rating of EOI or (1) a negative Initial Statement, (2) a negative Relationship, and (3) at least one Criticism based on either content or tone.

Three studies [41, 44, 45] adapted this by combining Criticism [6] and a rating of Warmth [47].

One study [40] utilised the Autism FMSS (AFMSS [48], coding system which includes four main components: (1) ratings of the quality of Initial Statement and (2) quality of Relationship (positive, neutral, or negative), (3) the Warmth and (4) EOI displayed (high, moderate, or low),as well as measuring a frequency count of Critical and Positive Comments. Overall EE status is determined by a combination of these components: High EE requires any of the four main components to be rated as negative/low, and there needs to be more Critical than Positive Comments; Moderate EE requires a rating of negative/low on a main component or more Critical than Positive Comments; Low EE describes all other cases. One study [39] combined components of the AFMSS and the FMSS-EE. One study [46] compared both the FMSS-EE and the AFMSS.

One study [42] chose to use the Preschool FMSS [49], which was developed for use when speaking about a child aged between 34 and 39 months old. The Preschool FMSS has four components: Initial Statement, Relationship, Warmth and EOI, with the frequency of critical and positive comments also being recorded. However, Serur et al. [42] used only the EOI score and a continuous Criticism score derived from the sum of all negative scores (negative Initial Statement, negative Relationship, low Warmth, and more negative than positive comments).

### Child Behavioural and Emotional Measures

The most commonly used measure of child/adolescent behavioural and emotional presentation across studies was the parent-report Child Behaviour Checklist (CBCL; [50]), which was used across six of the eight studies [39–42, 44–46] (Table 1). The CBCL is comprised of a problem behaviour scale and social competence scale. Within the problem behaviour scale there are 8 subscales: anxious, depressed, somatic complaints, thought problems, attention problems, rule-breaking behaviour, and aggressive behaviour. These subscales can be organised into two higher-order factors of internalising and externalising behaviours. A total behaviour score can be calculated by summing scores on all 8 subscales. Where appropriate, adapted versions were used according to the child age or primary language (1.5–5 year old version, [46], Hebrew version, [42]. One study [44] supplemented the information with the Teacher Rating Form of the CBCL. Scores were derived from the CBCL in a variety of ways, with two studies [39, 46] utilising the two higher-order factors of internalising and externalising behaviours, three studies utilising the total score [41, 44, 45], and one study opting to utilise total score alongside internalising and externalising behaviour scores [42]. Other outcome measures used included the Nosinger Child Behaviour Rating Form (NCBRF; [51]) used by Benson et al. [40], and the Behaviour Assessment System for Children – Parent Rating Scales (BASC2 PRS; [52] used by Zahka [43].

### Other Variables Measured

Seven of the eight included studies (all excluding [41] included measurements of additional variables that were analysed in relation to child behavioural and emotional outcomes (Table 1).

#### Autism Characteristics

Three studies included measures of general or specific autism characteristics in the child participants [39, 44, 46]. Hickey et al. [44] utilised the Social Responsiveness Scale (SRS-2 [53], to rate the severity of autism characteristics over the past 6 months, whilst Smith et al. [46] used the Calibrated Severity Scores from the Autism Diagnostic Observation Schedule (ADOS-2,[54]. Baker et al. [39] included a measure of child social competence through the subscale of the NCBRF.

#### Parental Psychopathology

Parental depression was measured in three studies [40, 45, 46] through the use of a range of measures including the Centre for Epidemiological Studies-Depression Scale (CES-D,[55], and the Depression Anxiety Stress Scale (DASS,[56]. Two studies also included a variable of parenting stress, Serur et al. [42] utilising the Parenting Stress Index (PSI [57], and Hickey et al. [45] using the Burden Interview [58].

#### Family/Parent Factors

Zahka [43] used the Cohesion scale of the Family Environment Scale (FES, [59]) to measure family cohesion. Smith et al. [46] included maternal education level in their analysis.

#### Child IQ and Cognitive Ability

Two studies [39, 43] included a variable of child IQ measured using the Stanford-Binet 5 Abbreviated Battery IQ (ABIQ; [60] or the Wechsler Intelligence Scale for Children (WISC-IV,[61]. One study [46] included a measure of child cognitive ability using the Mullen Scale of Early Learning Composite (MSEL,[62], while one study [44] included child intellectual disability status within their analysis.

### Quality of Studies

Three studies were rated as ‘good’ quality [41, 45, 46], whilst two studies were rated as ‘fair’ quality [39, 40], and three studies were rated as ‘poor’ quality [42, 43, 45]. The common cause of a lower rating was the lack of reporting regarding whether FMSS raters were blinded to participant group and small effect sizes. Hickey et al. [45] was rated ‘poor’ quality as the paper did not report whether FMSS raters were blinded, did not provide sufficient information to estimate effect sizes, and did not acknowledge within their worded results or discussion section the lack of acceptable fit statistics for their models. Zahka [43] was also rated as ‘poor’ quality due to a lack of clarity regarding the population sample, inclusion/exclusion criteria, and blinding of assessors. Due to the risk of bias within these two studies, the data extracted was given less weighting when synthesised with other included studies. Serur et al. [42] was rated as ‘poor’ due to the FMSS coding system selected being inappropriate as it is not designed for children over the age of 3.25 years while their population sample had a mean age of 5.35 years (Table 2).

**Table 2:** Quality appraisal of included studies

	Baker et al. [39]	Benson et al. [40]	Hickey et al. [41]	Hickey et al. [44]	Hickey et al. [45]	Serur et al. [42]	Smith et al. [46]	Zahka [43]
1. Was the research question or objective in this paper clearly stated?	Y	Y	Y	Y	Y	Y	Y	Y
2. Was the study population specified and defined?	Y	Y	Y	Y	Y	Y	Y	N
3. Was the participation rate of eligible persons at least 50%?	NR	NR	NR	Y	Y	NR	NR	NR
4. Were all the subjects selected or recruited from the same or similar populations (including the same time period)? Were inclusion and exclusion criteria for being in the study prespecified and applied uniformly to all participants?	Y	Y	Y	Y	Y	Y	Y	N
5. Was a sample size justification, power description, or variance and effect estimates provided?	Y	Y	Y	Y	N	Y	Y	Y
6. For the analyses in this paper, were the exposure(s) of interest measured prior to the outcome(s) being measured?	N	N	N	Y	Y	N	Y	N
7. Was the timeframe sufficient so that one could reasonably expect to see an association between exposure and outcome if it existed?	N	N	N	Y	Y	N	N	N
8. For exposures that can vary in amount or level, did the study examine different levels of the exposure as related to the outcome (e.g. categories of exposure, or exposure measured as a continuous variable)?	Y	Y	Y	Y	Y	Y	Y	Y
9. Were the exposure measures (independent variables) clearly defined, valid, reliable, and implemented consistently across all study participants?	N	Y	Y	Y	Y	Y	Y	Y
10 Was the exposure(s) assessed more than once over time?	N	N	N	Y	N	N	N	N
11. Were the outcome measures (dependent variables) clearly defined, valid, reliable, and implemented consistently across all study participants?	Y	Y	Y	Y	Y	Y	Y	Y
12. Were to outcome assessors blinded to the exposure status of participants?	Y	NR	Y	Y	NR	NR	NR	NR
13. Was loss to follow-up after baseline 20% or less?	NA	NA	NA	N	Y	NA	Y	NA
14. Were key potential confounding variables measured and adjusted statistically for their impact on the relationship between exposure(s) and outcome(s)?	Y	Y	Y	Y	Y	Y	Y	Y
Overall rating	Fair	Fair	Good	Good	Poor	Poor	Good	Poor
Additional comments	Exposure measure (FMSS) was not clearly defined; medium-large effect sizes	Small effect size	Medium-large effect sizes	Small-medium effect sizes	Effect sizes not reported; models do not have adequate fit statistics; small effect sizes	Coding system was not appropriate for sample population age. Small-medium effect sizes	Medium-large effect sizes	Specificity of population sample is unclear; unclear if inclusion and exclusion criteria were predefined; no reference to whether outcome assessors were blinded to exposure status

### Relationship Between EE and Child Behavioural/Emotional Outcomes

#### Overall EE

Of the three studies which included overall EE within their analysis only one found a significant relationship to child behavioural scores. Smith et al. [46] found that overall EE, as measured by the AFMSS, predicted concurrent and subsequent child internalising and externalising behaviours at baseline and follow-up, however they found no association between overall EE measured by the original FMSS (FMSS-EE) and child internalising or externalising behaviour scores. Similarly, Zahka [43] found no significant effect of overall FMSS-EE on child internalising or externalising behaviour. In contrast to Smith et al. [46], Benson et al. [40] found no significant correlation between overall AFMSS EE and child behaviour scores, however they only measured overall behaviour scores and were using a primary school age population of children, whilst Smith et al. [46] utilised a preschool age population (Table 3). Therefore, whilst overall FMSS-EE showed no relationship to measures of child behaviour, the AFMSS EE displays a relationship to child behaviour scores when distinguishing between internalising and externalising within preschool aged children. However, given that only one of the three studies produced significant relationships, it may be the case that overall EE is not a particularly useful contrast in understanding the impact of parental attitudes on child behaviour.

**Table 3:** Analysis, results and main limitations of studies

First author (year)	Data analysis	Relationship between parental EE and child/adolescent emotional/behavioural presentation (effect size)	Relationships between additional variables and child/adolescent behavioural/emotional outcomes (effect size)	Main limitations
Baker et al. [39]	Correlations, hierarchical regressions, Simple slope computations	Criticism was positively correlated with externalising behaviour (*r* = 0.48) Warmth was negatively correlated with externalising behaviour (*r* = -0.36) Criticism significantly predicted externalising behaviour at all steps of a hierarchical regression model ($\beta =$ 0.32–0.51)	Child IQ was not significantly related to internalising or externalising behaviour problems Child autism characteristic severity was not significantly correlated to child internalising or externalising behaviours	Sample size was generally appropriate for regression analyses but modest for investigating interactive effects No comparison group of neurotypical children Child measures were measured through parent report, observational measure may reduce potential report bias Only focused on primary care giver, rather than including both parents in two-parent households
Benson et al. [40]	Correlations, regressions	Overall EE and child problem behaviour were not found to be significantly correlated Warmth was positively associated with child problem behaviour ($\rho$ = 0.24), however no other individual AFMSS components were found to be significantly correlated to child problem behaviour Overall EE was not found to significantly predict severity of child problem behaviour once other child, parent, and family factors were included in the model	Maternal depression had significant unique predictive value for overall child problem behaviour (($\beta = .0337).$	Construct and concurrent validity of the AFMSS were not assessed As the data was collected concurrently, the direction of effects between EE and parent, child, and family variables cannot be determined As the measures were based on parent report, the observed associations may be artificially inflated due to shared method variance Only maternal EE was explored The sample consisted of most white, well-educated, and economically advantaged mothers who volunteered for the study, therefore may not be generalisable to other populations
Hickey et al. [41]	Phi coefficients, latent class analysis, MANOVAs	Parents who displayed high warmth and low criticism towards their child reported lower levels of child behavioural and emotional problems, whereas parents who displayed low warmth and high criticism towards their child reported higher levels of child behavioural and emotional problems (${\eta }_{p}$ ^2^ = 0.18 and 0.10 for mothers and fathers reports respectively)		No comparison group, therefore it is not possible to determine whether these differences may also occur in families of typically developing children, or those with children with other conditions The families used in the sample were fairly homogeneous
Hickey et al. [44]	T-tests, multiple linear regression, structural equation modelling	Significant correlations were found between: Mother warmth at baseline and child behavioural/emotional problems at baseline, 12-month follow-up and 24-month follow-up (*r* = -0.23, -0,30, -0.23 respectively); Mother warmth at 24-month follow-up and child behaviour at 24-month follow-up (*r* = -0.29); Father warmth at 24-moth follow-up and child behaviour at 24-month follow-up (*r* = -0.28); and Mother criticism at baseline and child behavioural/emotional problems at 24-month follow-up (*r* = 0.38), and between mother criticism at 24-month follow-up and child behaviour at 24-month follow-up (*r* = 0.28) Bidirectional associations were present between parental EE and child behavioural/emotional problems: Mother warmth at baseline predicted child behavioural/emotional problems at 12-month follow-up ($\beta = -0.27).$ Child behavioural/emotional problems at 12-month follow-up predicted father warmth at 24-month follow-up ($\beta = -0.23)$ and mother criticism at 24-month follow-up ($\beta =0.22).$ Mother warmth at 24-month follow-up* (*$\beta = -0.28)$ and father criticism at 24-month follow-up ($\beta = 0.29)$ predicted child behavioural/emotional problems at 24-month follow-up	Child autism characteristic severity was positively correlated with child behaviour problems at baseline (*r* = 0.64), 12-month follow-up (*r* = 0.67), and 24-month follow-up (*r* = 0.61) Severity of autism characteristics at Time 1 was correlated with behaviour at Time 1 (*r* = 0.64), Time 2 (*r* = 0.43), and Time 3 (*r* = 0.41), characteristic severity at Time was correlated with behaviour at Time 2 (*r* = 0.67) and Time 3 (*r* = 0.43). Characteristic severity at Time 3 was correlated with behaviour at Time 3 (*r* = 0.61)	Sample as fairly homogenous in race/ethnicity and socio-economic status Time points only spanned middle-childhood. Unable to make conclusions about the longer-term directional relationships between parent–child relationships and child functioning or how these look at other developmental stage or shift over time
Hickey et al. [45]	Correlations, t-tests, structural equation modelling	Each parent’s warmth was significantly negatively correlated with their own ratings of child behaviour problems (mother: *r* = -0.25; father: *r* = -0.26) Father warmth was negatively correlated with mother rating of child behavioural problems (*r* = -0.21) Mother criticism was significantly positively correlated with mother rating of child behavioural problems (*r* = 0.28)	Mother level of parenting stress was significantly positively correlated with mother and father ratings of child behavioural problems (*r* = 0.52, 0.28 respectively) Father level of parenting stress was significantly positively correlated with mother and father ratings of child behavioural problems (*r* = 0.36, 0.53 respectively) Parent level of depressive symptoms was significantly positively correlated with their rating of child behavioural problems (mother: *r* = 0.31; father: *r* = 0.33)	Homogenous sample in terms of race/ethnicity and middle-upper socio-economic status
Serur et al. [42]	ANOVAs, correlations, t-tests, regression analyses	Criticism was significantly positively correlated with internalising behaviour (*r* = 0.44), externalising behaviour (*r* = 0.29), and overall behaviour (*r* = 0.42), but criticism was not found to be predictive at regression analysis EOI predicted child externalising ($\beta =0.30)$, and total behaviour problems ($\beta =0.21).$	Parenting stress was correlated with child internalising (*r* = 0.61), externalising (*r* = 0.38), and overall (r = 0.38) behaviour problems and had significant predictive value for internalising ($\beta =0.51)$, externalising ($\beta =0.38)$, and total ($\beta = 0.38)$ behaviour problems	Families of children with 22q11DS came from lower SES backgrounds compared with families of children with autistic and TD children PFMSS coding system was used despite not being designed for children of the sample population’s age
Smith et al. [46]	ANOVAs, correlations, hierarchical regression	FMSS measures were found to be largely unrelated to child behaviours AFMSS EE predicted child internalising at baseline ($\beta = 0.33)$ but not follow-up, and externalising behaviours at both baseline and follow-up ($\beta = 0.28, 0.34$ respectively) AFMSS Warmth was associated with baseline internalising behaviour (${\eta }_{p}$ ^2^ = 0.14) and follow-up externalising behaviour (${\eta }_{p}$ ^2^ = 0.17) AFMSS Critical Comments was predictive of child externalising behaviours at baseline ($\beta = 0.31).$ AFMSS Moderate Warmth was found to predict fewer child internalising problems at baseline ($\beta = -0.35$). AMFSS Low Warmth predicted greater externalising behaviour at follow-up ($\beta =0.30).$	Child autism characteristic severity was positively correlated with internalising behaviours at baseline (*r* = 0.31), but not at follow-up or for externalising behaviour. Characteristic severity had significant unique predictive value for baseline internalising behaviour ($\beta = 0.28).$ Parental psychopathology was correlated with baseline externalising behaviour (*r* = 0.28), and follow-up internalising (*r* = 0.38) and externalising (*r* = 0.30) behaviour problems, but was only predictive of internalising behaviour at follow-up ($\beta = 0.34).$ Child cognitive ability was negatively correlated to internalising behaviour at baseline (*rs* = − 0.33) and follow-up (*rs* = − 0.32) and was predictive of internalising behaviour at follow-up ($\beta = - 0.28).$ Maternal education carried significant unique predictive value for baseline internalising behaviour ($\beta = - 0.40).$	Relatively small sample size prevented the investigation of interacting effects in the regression models Possibility of common-rater bias as both the FMSS/AFMSS and measures of child psychopathology were based on perceptions of the same parent
Zahka [43]	MANCOVAs, hierarchical regressions	Parental EE did not moderate the significant difference in externalising behaviours seen between the HFA and comparison group Neither parental EE nor the effect of the interaction between diagnostic group and parental EE were significant in the model for internalising behaviours	Family cohesion was found to have significant predictive value for externalising behaviours of aggression ($\beta = - 0.23)$ and hyperactivity ($\beta = - 0.20).$	Limited variability in EE status may have reduced the possibility of detecting differences By using only overall EE, an important difference between criticism and emotional over-involvement may have been masked As the data was collected concurrently, the direct of effects cannot be clarified Several analyses were approaching significance which may suggest that the sample size was not large enough to gain adequate power given the high number of analyses being conducted and the required post hoc corrections

#### Criticism

Four of the five studies which included direct analysis of criticism and child behavioural presentations found significant relationships (Table 3). In three studies, criticism was found to have a significant positive correlation with overall child behavioural problems [42, 44, 45], whilst two studies found positive correlations between criticism and externalising behaviour [39, 42]. One of these two studies [42] also found a significant positive correlation between criticism and internalising behaviour, however this was not supported by the findings of Baker et al. [39] who found no significant correlation with internalising behaviour. Three of the studies finding significant correlations included further analysis using either hierarchical regression or structural equation modelling, with Hickey et al. [44] finding that maternal criticism at baseline and 24-month follow-up was predictive of child overall behaviour at 24-month follow-up, and Baker et al. [39] finding that criticism significantly predicted child externalising behaviour. However, Serur et al. [42] did not find criticism to have significant predictive value within a regression model. In contrast to the previous four studies, Smith et al. [46] did not find any significant correlations between child internalising or externalising behaviour at baseline or follow-up, however they were utilising a population of children at preschool-age whilst all other studies were using populations of primary school-aged children, suggesting that criticism may not have as much impact upon younger children.

Of the two studies that included separate analyses of paternal criticism and maternal criticism one found no significant correlation between paternal criticism and child behaviour scores when using a single time point [45], whilst one found that paternal criticism at 24-month follow-up was predictive of child behaviour problems at 24-month follow-up [44], suggesting that mothers’ expressions of criticism may have a greater initial impact on child behaviour than fathers’, but if paternal criticism is prolonged it may begin to impact child behaviour.

One study [44] included an analysis of bidirectional relationships and found that child behaviour scores at 12-month follow-up predicted maternal criticism at 24-month follow-up, suggesting that there is a reciprocal process occurring between mother and child which have significant influence upon each other’s emotional and behavioural responses.

#### Warmth

The five studies that included direct analysis of warmth and child behaviour scores all found significant relationships (Table 3). The four of the five studies found a negative association, where increased parental warmth was associated with decreased behaviour scores. Of the three studies measuring overall child behaviour scores, two found a negative correlation between FMSS-EE warmth and behaviour scores [44, 45] and in one study [44] this relationship was maintained across time with maternal warmth at baseline being negatively correlated with child behaviour scores at baseline, 12-month and 24-month follow-up. Maternal warmth at baseline was also found to predict child behaviour scores at 12-month follow-up, whilst maternal warmth at 24-month follow-up predicted child behaviour scores at 24-month follow-up. In contrast, Benson et al. [40] found a positive association between overall child behaviour scores and maternal warmth when measured by the AFMSS, where increased maternal warmth was associated with increased child behaviour scores.

This finding is conflicting with Baker et al. [39] who found that warmth, as measured by the AFMSS, was negatively correlated with child externalising behaviour problems. However, warmth was not found to have significant predictive value within a regression analysis and no relationship was found to internalising behaviours. Another study utilising the AFMSS [46] found further conflicting results in negative associations between warmth and child internalising behaviour at baseline, and externalising behaviour at follow-up. These associations were maintained in regression analyses which found moderate warmth to be predictive of lower child internalising behaviour at baseline, and low warmth to be predictive of greater child externalising behaviours. Given these varying results across AFMSS studies, the evidence of a relationship between AFMSS warmth and child behaviour is currently inconclusive, however FMSS-EE maternal warmth may relate to, and predict, current and future child behaviour problems.

Two studies included analysis of paternal warmth, both finding significant relationships between paternal warmth and child behaviour. Hickey et al. [45] found a significant negative correlation between paternal warmth and child behaviour scores, while Hickey et al. [44] found a similar significant negative correlation between paternal warmth and child behaviour scores at 24-month follow-up, but not at baseline or 12-month follow-up. When exploring bidirectional associations, they found that child behaviour scores at 12-month follow-up predicted paternal warmth at 24-month follow-up. These findings suggest that fathers may have an important impact on child behaviour through their expression of warmth towards their child, and that fathers and their children can have a reciprocal impact upon each other.

#### Combinations of Criticism and Warmth

Hickey et al. [41] explored the various possible combinations of maternal and paternal warmth and criticism that may be expressed by a heterosexual parenting couple. They found that lower ratings of child behaviour problems were associated with parenting couples where both parents expressed low criticism and high warmth, when compared to couples where both parents expressed high criticism and low warmth, and couples where the mother expressed low warmth and the father expressed low criticism (Table 3). These findings further support the significant relationship between parental criticism and warmth and child behavioural presentations.

#### Additional EE Variables

Of the three studies measuring EOI, only one found a significant relationship (Table 3). Serur et al. [42] found EOI, as measured by the PFMSS coding system, was positively correlated with internalising, externalising, and total child behaviour scores, and that it was predictive of externalising and total behaviour scores. However, the children within this sample population were older than recommended for the use of the PFMSS, therefore this finding must be taken with caution. When using the original FMSS coding system, Smith et al. [46] found no significant association between EOI and child behaviour. When using the AFMSS coding system Benson et al. [40] found a similar lack of association, whilst Smith et al. [46] was unable to conduct an analysis due to AFMSS EOI ratings having too limited a range in scores. These findings suggest that child behaviour is relatively unrelated to EOI and that results may be dependent on the appropriateness of the coding system used.

Two studies [40, 46] measured four additional variables: initial statement, relationship, positive comments, and critical comments. While both studies found no significant relationship between positive comments and child behaviour scores, their results were contradictory for all other variables, with Benson et al. [40] finding no significant relationship between any AFMSS EE variables and child behaviour, and Smith et al. [46] finding significant relationships to child externalising behaviour in AFMSS initial statement, relationship, and critical comments, and FMSS-EE initial statement. The AFMSS initial statement and relationship were found to have a significant association to child externalising behaviour at follow-up, whilst AFMSS critical comments were found to have a positive correlation with externalising behaviours at both baseline and follow-up, and was predictive of externalising behaviours at baseline. However, the FMSS-EE was found to have a significant association with, and was predictive of, externalising behaviour at follow-up. No significant relationships were found between internalising behaviour and initial statement, relationship, positive comments, or critical comments measured by the AFMSS or the original FMSS. These conflicting results suggest that further exploration of these four variables is needed to gain a clearer picture of their relationship to, and predictive value for, child behaviour problems.

### Relationships Between Additional Variables and Child Behavioural/Emotional Outcomes

#### Autism Characteristics

Three studies included analysis exploring the relationship between child autism characteristics and child behavioural presentations, with varying results (Table 3). Hickey et al. [44] found significant positive correlations between child autism characteristic severity and overall child behaviour problems at baseline, 12-month follow-up and 24-month follow-up. These relationships showed stronger correlations than those found between EE components and child behaviour. Smith et al. [46] found a similar significant positive correlation between characteristic severity and baseline internalising behaviour and characteristic severity was found to carry significant predictive value for baseline internalising behaviour, however no significant associations were found to internalising behaviour at follow-up or externalising behaviour at baseline or follow-up. Within their regression analysis AFMSS-EE was found to have greater unique predictive value than autism characteristic severity. Meanwhile, Baker et al. [39] found no significant correlations between autism characteristic severity and internalising or externalising behaviour. Given the variability in results, the impact of child autism characteristics on child behaviour problems appears unclear and may warrant further investigation, however a recent systematic review and meta-analysis identified both positive and negative correlations between behaviour problems and specific autism characteristics [63].

#### Parental Psychopathology

Four studies explored the relationship between parental psychopathology and child behaviour presentations, with all four studies reporting significant associations (Table 3). Both Serur et al. [42] and Hickey et al. [45] found a positive correlation between parenting stress and child behaviour problems, and in a regression analysis Serur et al. [42] found parenting stress to be predictive of child internalising, externalising, and overall behaviour problems. Both studies found that parenting stress had stronger correlations than EE components did, and Serur et al. [42] found parenting stress to have greater unique predictive value than EE components. Similar patterns were observed in parent depressive symptoms with both Smith et al. [46] and Hickey et al. [45] finding a significant positive correlation between parent depressive symptoms and child behaviour problems, and parent depressed mood being identified as a significant predictor of overall child behaviour problems [46] and child internalising behaviour at follow-up [40]. Within these studies parent depressive symptoms were found to have stronger correlations to, and have greater unique predictive value for, child problem behaviour than AFMSS-EE. The consistency of these results suggest that parent psychopathology can play an important role in child behaviour and may be a worthwhile area to explore in future intervention studies aimed at behaviour problems in autistic children.

#### Child IQ and Cognitive Ability

Three studies explored the relationship between child IQ/cognitive ability and their behavioural presentations (Table 3). Smith et al. [46] found that child cognitive ability was correlated with internalising behaviour at baseline and follow-up and was predictive of child internalising behaviour at follow-up with greater unique predictive value than AMFSS-EE. These findings were not supported by Baker et al. [39] or Zahka [43] both of whom found that child IQ was unrelated to internalising or externalising behaviours. These findings suggest child IQ/cognitive ability may not have a significant role in the behavioural presentations of autistic children, however further research may be required to provide further evidence.

#### Family/Parent Factors

Two studies explored additional family or parent factors (Table 3). Smith et al. [46] included analysis of the role of maternal education level and found it to be associated with internalising and externalising behaviours at both baseline and follow-up, and was predictive of baseline internalising behaviour with greater unique predictive value than AFMSS-EE. Zahka [43] included a measure of family cohesion and found this to be predictive of child externalising behaviour of aggression and hyperactivity whilst EE was not found to be related to these outcomes. These findings suggest that there may be important elements of parent demographics and wider family dynamics that could warrant further exploration in understanding child behaviour problems.

## Discussion

This review intended to address the limitations of previous systematic reviews into the relationship between parental EE and behavioural presentations of autistic children and adolescents by synthesising results of studies utilising a ‘gold standard’ measure of autism, only using FMSS measures of EE, and maintaining a child and adolescent age range. Seven of the eight studies found a significant relationship between one or more FMSS variables and child behavioural scores. Three additional variables showed evidence of greater predictive value than EE for child behaviour outcomes.

The studies summarised within this review show consistent links between parental EE and child behavioural presentations, however the findings appear to be variable across coding systems. The AFMSS yielded more predictive interactions for overall EE, but produced mixed results for parental warmth and criticism. When measured by the AFMSS, overall EE was found to predict internalising and externalising child behaviours in pre-school age children but not primary school-aged children, however, when measured by the FMSS-EE, these same interactions were not observed.

When measured by the FMSS-EE parental criticism was found to correlate to, and be predictive of overall child behaviour problems, however when measured by the PFMSS a similar correlational relationship was found but criticism was not significantly predictive of behaviour problems. Studies utilising the AFMSS produced mixed results, with one showing similar positive correlations, and significant predictive value for externalising behaviour problems, whilst another showed no significant relationship, however this could be due to differences in the population samples’ ages, suggesting that criticism may be a useful predictor of child behaviour outcomes across coding systems.

When measured by the adapted FMSS-EE, maternal warmth was shown to be negatively correlated with child behaviour, and this relationship was maintained at regression in one study, showing warmth to be predictive of future decreases in behaviour. However, the AFMSS yielded varying results of relationships between parental warmth and child behaviour, with differences across studies in the direction of association and predictive value of warmth in regression analyses.

These differences in relationships across coding systems highlight a current issue in the use of FMSS in developmental research. Whilst significant efforts have been made to design alternative coding systems that are more appropriate for specific populations, particularly preschool age children and autistic children, there are now at least five distinct coding systems and at least two additional extended coding systems [7], creating a potential dilemma for researchers in selecting the most appropriate system for their investigations. This dilemma may be further complicated if a participant sample were to cross multiple characteristics, for example focusing on preschool age autistic children. In addition, there is a marked difference in researcher’s approaches to utilising the FMSS measurements even within those employing the same coding system. Some researchers opt to analyse overall EE scores and each subcomponent, whilst others choose to only analyse the overall score, or only selecting specific subcomponents of interest. This variability in coding systems and approaches to selecting measurements for analysis creates substantial difficulties when synthesising findings across studies due to the significant heterogeneity across studies, which creates limitations for the review. It may be beneficial for future FMSS research to work towards developing ‘gold standard’ coding systems and for a unified approach to analysing measurements within these coding systems.

Despite these challenges in synthesising findings across coding systems, a consistent finding of significant relationships between maternal criticism and child behaviour was identified, with evidence towards positive correlations between internalising, externalising, and overall child behaviour, and some evidence towards criticism being a significant predictor of child overall and externalising behaviour. These findings reflect those found in research into typically developing children [15], and autistic individuals across a broader age range [29], suggesting that the experiences of maternal criticism may have more universally adverse effects to the behavioural development of all individuals, and that a more universal theoretical framework, such as Bronfenbrenner’s [64] ecological systems theory or Bridgett et al.’s [65] self-regulation intergenerational transmission model, may be more appropriate for understanding the impact of parental attitudes. These similarities in findings across neurodevelopmental groups and ages suggest that parenting interventions aimed at reducing maternal criticism (e.g. [66] may be appropriate for mothers of autistic and neurotypical children and at varying ages.

Whilst previous research has been largely focused on the relationship between mothers and their children, two studies within this review provided interesting findings regarding the role of fathers in parent–child dynamics. Whilst no significant relationships were found for paternal criticism, paternal warmth was found to have a significant negative correlation to child behaviour problems. These findings may suggest that the combination of low maternal criticism and high paternal warmth may create a family environment which is the most protective against the development of behavioural problems. However, combinations of maternal and paternal level of EE were explored by one study [41] which found that lower behaviour scores were associated with family environments where both parents displayed low criticism and high warmth. Given this preliminary evidence of paternal EE having an important role in child behaviour presentations, interventions aimed at improving child behaviour through addressing parental EE should be making concerted efforts to include fathers of autistic children.

Historically, research around parental EE has focused on the potential impact of EE on a range of psychiatric, health, and behavioural outcomes of young and adult children. However, transactional [67], attachment and social learning [68] theories of child development, would suggest that the parent–child relationship is both influenced by child behaviour, and is key to shaping child behaviour across time, creating a reciprocal relationship between parental emotional expression towards the child, and the child’s behaviour. It is likely that the impact of the reciprocal relationships is heightened in families of autistic children due to increased difficulties in social-relatedness [69]. Preliminary evidence from one study within the review [44] lends support to these theoretical perspectives as bidirectional relationships were found between child behaviour scores and parental EE. Maternal warmth was found to be predictive of future child behaviour scores, whilst child behaviour scores were found to be predictive of future maternal criticism and paternal warmth.

One area that remain contentious in the use of FMSS EE coding systems is the inclusion of the emotional over-involvement (EOI) subcomponent as many researchers argue that EOI is not associated with features of observed parent–child interactions [7]. The findings of this review support the concerns raised within these debates, as results across studies were variable, with only the PFMSS producing significant relationships between EOI and child behaviour in one study, and the range of scores being too small to conduct analysis for AFMSS within the same study [46]. Whilst two studies included analysis of four additional EE variables, the results were conflicting and further research would be necessary to draw any conclusions on their role in child behavioural presentations.

A range of additional child and parent variables were explored across studies which provide useful insights into potential factors that could play an added role in the development of child behaviour problems. In line with previous research [70, 71], parenting stress and depressive symptoms were shown to have consistent relationships with, a predictive value for, child behaviour problems across studies. Given that these relationships were found to be stronger than those for EE, addressing parental stress and depression may be an important area for future interventions aimed at reducing child behaviour problems. Another relatively consistent finding across studies was the lack of association between child cognitive ability or IQ and their behavioural presentations, suggesting that interventions aimed at reducing child behaviour problems may be suitable for families of autistic children across a range of intellectual abilities. Findings relating to the severity of child autism characteristics were variable and may need further exploration to establish a clearer picture to inform future clinical intervention efforts. Two additional variables that may warrant further investigation are maternal education level and family cohesion as both were found to have predictive value for child behaviour, but results were limited to only one study each.

The results of this review provide promising evidence of the role of parental EE in the development of behavioural difficulties in autistic children, and the potential bidirectional nature of this relationship, which is supported by current child development theories. Given the greater consistency of results for the predictive value of parental criticism, and the preliminary evidence for the importance of paternal warmth, in child behaviour outcomes, future directions for FMSS research should explore the impact of interventions designed to reduced parental criticism and increase parental warmth, as these may having promising results for reducing child behaviour problems across a range of intellectual abilities and age ranges. However, given the evidence of bidirectional relationships and additional factors of parental stress and psychopathology, future interventions would likely be more effective if they were to target multiple outcomes instead of focusing on focusing on purely parent-, or child-directed interventions. For example, De Clercq et al. [72] suggests a combination of psychoeducation to alter parental perceptions of their children, accompanied by skills training, problem-solving and communication techniques, with the potential for additional family interventions addressing emotion regulation strategies as a means for addressing multiple contributory factors. Careful consideration should be given to how EE coding systems are used within future research to increase homogeneity across studies, creating more opportunities for valuable systematic and meta-analytic reviews in the future.

## Summary

Parental criticism and warmth may be useful predictors of child behaviour problems in autism, however evidence suggests parenting stress and parental psychopathology may also play an important role. Selection of FMSS coding systems is inconsistent in autism research and future research would benefit from developing a unified approach. The relationship between parental EE and child behaviour outcomes appears to be similar to that seen in research in neurotypical children, therefore interventions may be appropriate for families both autistic and neurotypical children. Interventions may be most effective when taking a multi-dimensional approach addressing child, parent, and child-parent relationship factors.

## Data Availability

Data is available upon reasonable request.
